# *Dscam1* in Pancrustacean Immunity: Current Status and a Look to the Future

**DOI:** 10.3389/fimmu.2017.00662

**Published:** 2017-06-09

**Authors:** Sophie A. O. Armitage, Joachim Kurtz, Daniela Brites, Yuemei Dong, Louis Du Pasquier, Han-Ching Wang

**Affiliations:** ^1^Institute for Evolution and Biodiversity, University of Münster, Münster, Germany; ^2^Tuberculosis Research Unit, Swiss Tropical and Public Health Institute, Basel, Switzerland; ^3^Zoological Institute, University of Basel, Basel, Switzerland; ^4^Department of Molecular Microbiology and Immunology, Bloomberg School of Public Health, John Hopkins University, Baltimore, MD, United States; ^5^Department of Biotechnology and Bioindustry Sciences, College of Bioscience and Biotechnology, National Cheng Kung University, Tainan, Taiwan

**Keywords:** alternative splicing, crustaceans, isoform diversity, immunoglobulin domain, innate immunity, insects

## Abstract

The *Down syndrome cell adhesion molecule 1* (*Dscam1*) gene is an extraordinary example of diversity: by combining alternatively spliced exons, thousands of isoforms can be produced from just one gene. So far, such diversity in this gene has only been found in insects and crustaceans, and its essential part in neural wiring has been well-characterized for *Drosophila melanogaster*. Ten years ago evidence from *D. melanogaster* showed that the *Dscam1* gene is involved in insect immune defense and work on *Anopheles gambiae* indicated that it is a hypervariable immune receptor. These exciting findings showed that *via* processes of somatic diversification insects have the possibility to produce unexpected immune molecule diversity, and it was hypothesized that *Dscam1* could provide the mechanistic underpinnings of specific immune responses. Since these first publications the quest to understand the function of this gene has uncovered fascinating insights from insects and crustaceans. However, we are still far from a complete understanding of how Dscam1 functions in relation to parasites and pathogens and its full relevance for the immune system. In this Hypothesis and Theory article, we first briefly introduce *Dscam1* and what we know so far about how it might function in immunity. By focusing on seven questions, we then share our sometimes contrasting thoughts on what the evidence tells us so far, what essential experiments remain to be done, and the future prospects, with the aim to provide a multiangled view on what this fascinating gene has to do with immune defense.

## Introduction

### *Dscam1*: Mutually Exclusive Alternative Splicing Generates Isoform Diversity

There are few genes that encode for such extreme molecular diversity as *Dscam1*, the insect and crustacean homolog of the human Down syndrome cell adhesion molecule (DSCAM) (1, 2). Although *Dscam1* shares homology with DSCAM, only *Dscam1* has evolved the possibility to produce a profusion of protein isoforms. Since *Dscam1* was discovered as a cell surface hypervariable axon guidance receptor in *D. melanogaster* (2), our knowledge of its functions in the nervous system and the immune system as well as its evolution across insects and crustaceans (i.e., the subgroup of the arthropods that is called Pancrustacea) has expanded extensively [reviewed in Ref. (3–10)], yet we are still far from a complete understanding of how Dscam1 reacts and responds to parasites and pathogens.

**Figure 1:**
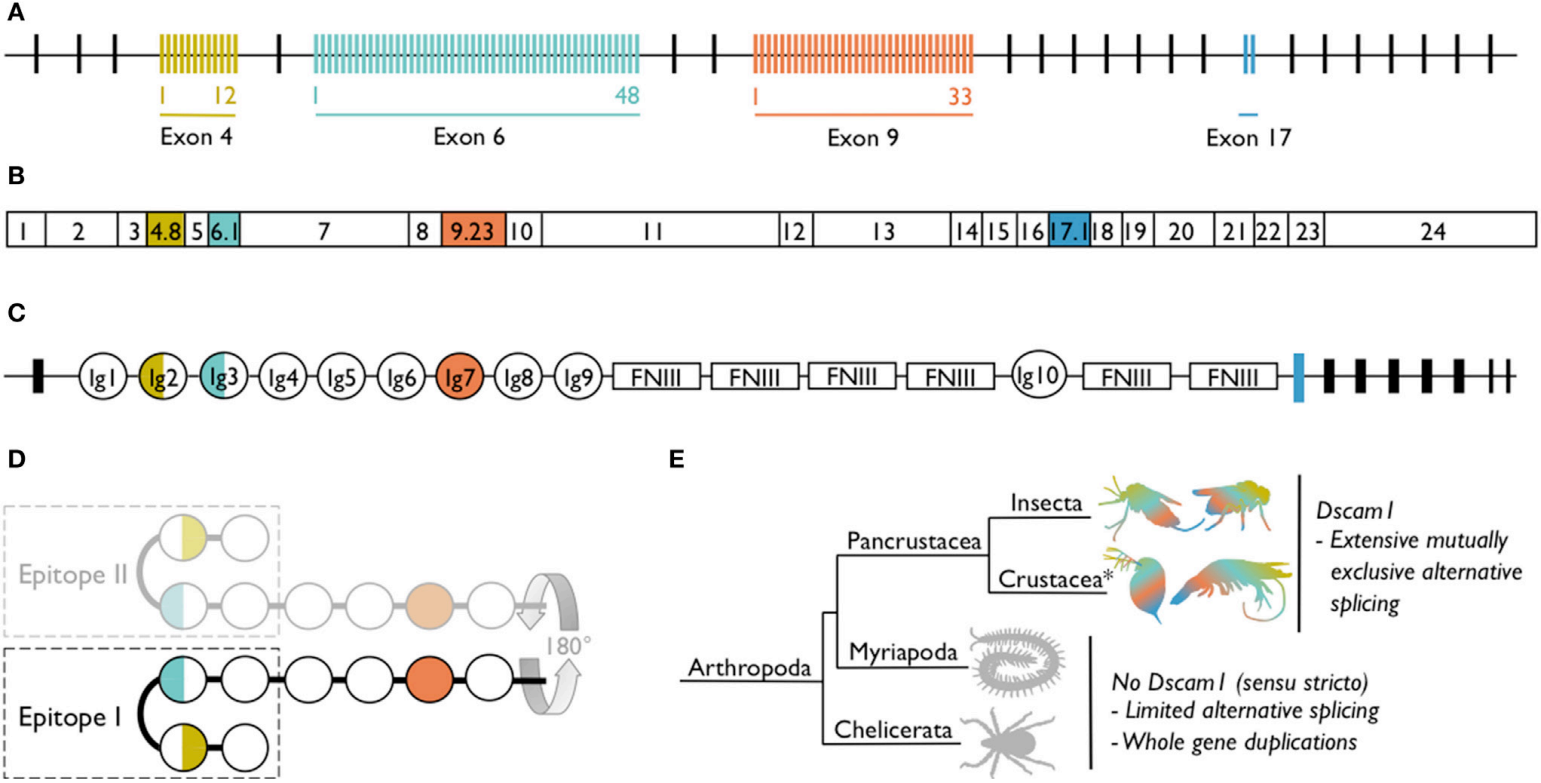
*Dscam1* in *Drosophila melanogaster* and known occurrence of *Dscam1* in arthropods. **(A)** *D. melanogaster Dscam1* genomic DNA structure contains 20 constant exons (black lines). Four exon clusters contain variable numbers of alternative exons (colored lines): exon 4 contains 12, exon 6 contains 48, exon 9 contains 33, and exon 17 contains 2 variants. **(B)** *Dscam1* mRNA contains every constant exon (white boxes), but through the process of mutually exclusive alternative splicing, only one of each of the alternative exons is present in each mRNA; one exon combination for *D. melanogaster* is illustrated. **(C)** Dscam1 protein structure, where Ig indicates an immunoglobulin domain and FNIII indicates a fibronectin type III domain. The alternatively spliced exons encode the N-terminal halves of Ig2 and Ig3, all of Ig7, and the transmembrane domain. **(D)** Ig1 to Ig4 form a horseshoe configuration (24). Epitope I is one side of the horseshoe and in the nervous system engages in homophilic binding with identical Dscam1 isoforms coded for by the identical exon 4, 6, and 9 variants; the other side of the horseshoe, epitope II, has been proposed to bind to non-Dscam1 ligands, i.e., pathogen-related ligands. [**(A–D)** after (16)]. **(E)** *Dscam1* as illustrated in **(A–C)** has, to date, only been found in pancrustaceans. Myriapods and chelicerates have diversified the *Dscam* gene family *via* other routes. *Crustacea is considered a paraphyletic group containing the hexapods; phylogeny follows Legg et al. (17).

*Dscam1* [synonymous with *Dscam, Dscam-hypervariable* (*Dscam-hv*) and species-specific notations, e.g., the shrimp *Litopenaeus vannamei Dscam1* has been named *LvDscam*] has a complex gene structure whereby clusters of alternative exons encode for different immunoglobulins (Igs) domains (Figure 1). As an example, in *D. melanogaster* exons 4, 6, and 9 have numerous alternative sequences (2). Exon 4 has evolved 12 alternative variants, exon 6 has 48 [of which 47 are transcribed (11–13)], and exon 9 has 33 variants (Figure 1A). The number of alternative variants is not conserved across species, but the existence of multiple variants within three exon clusters is consistent across all pancrustaceans studied to date. However, in species other than *D. melanogaster*, the orthologous exon clusters sometimes have different numbering [e.g., exons 4, 6, and 10 in *Anopheles gambiae* (14)] because of differing positions of exon–exon boundaries. The pre-mRNA undergoes mutually exclusive alternative splicing, so that each mRNA contains only one of the possible variants from each of the three alternative exon clusters (Figure 1B). Across species, the alternatively spliced exons code for the N-terminal halves of Ig2 and Ig3 and the whole of Ig7 (Figure 1C). These Ig domains are located in the extracellular portion of the protein. Mutually exclusive alternative splicing of the exons encoding the extracellular region, could potentially lead to the production of 12 × 48 × 33 = 19,008 gene isoforms (18,612 if the non-transcribed exon in cluster 6 is excluded). If exon 17, which has two alternatively spliced variants and encodes the transmembrane region of the protein, and exons 19 and 23, which can be contained within or skipped from the cytoplasmic region of the protein (15), are included in the isoform diversity calculation, the estimate increases to just under 150,000 gene isoforms. This is an incredible amount of diversity to be expressed by just one gene.

### Involvement in the Nervous System

Our knowledge about Dscam1’s function in the nervous system comes predominantly from research on *D. melanogaster*, where it has been extensively reviewed [e.g., Ref. (3–5, 9, 16, 18, 19)]. The diverse extracellular domains of the Dscam1 protein facilitate its function as a molecular surface code, which enables neurites to tell self from non-self, thus avoiding neuronal self-connectivity (5). Homophilic binding, i.e., binding between identical isoforms and subsequent repulsion, is the key to Dscam1’s function in self-recognition. In brief, expression analyses have estimated that individual cells produce a much reduced portion of the total number of possible isoforms, i.e., in the order of tens of isoforms, and that cells produce a suite of isoforms that are different to their neighboring cells (11). These two points are important because they make it highly likely that sister neurites from the same neuron will express identical Dscam1 isoforms that will in turn differ from the neighboring cells; identical isoforms will bind to each other, but not at all, or only weakly, to non-identical isoforms (20, 21). Once identical isoforms have interacted, the protein’s endodomain (cytoplasmic tail) converts isoform recognition into repulsion between the sister neurites and promotes self-avoidance (22). In the nervous system the identity of each isoform, i.e., the combination of exons 4, 6, and 9 that encode it, does not matter, but it is essential that neighboring neurons express different isoforms from one another (23). Non-self recognition is thereby effectively a game of probabilities, where the number of potential Dscam1 isoforms and the number of and stochasticity with which cells express Dscam1 isoforms determine the rules. In contrast to the nervous system, if Dscam1 diversity affords the immune system the ability to discriminate between different pathogens, it is hypothesized that the identity of individual isoforms does matter.

### One Protein, Two Roles?

Elucidation of the protein structure of *D. melanogaster* Dscam1 hinted at how one protein might function in both the nervous system and the immune system. *D. melanogaster* Dscam1 Ig1 to Ig4 form a horseshoe configuration, with an independent interaction surface on either side of the horseshoe (24). Some of the amino acids encoded for by exons 4 and 6 can be found on either one (epitope I) or the other (epitope II; Figure 1D) interaction surfaces. By swapping peptide segments, Meijers et al. (24) found that it is epitope I that engages in homophilic binding specificity, whereas epitope II was hypothesized to bind to non-Dscam1 ligands, i.e., heterophilic binding. It has not to date been demonstrated empirically that epitope II binds to non-Dscam1 ligands, but it has been hypothesized that the ligands could be antigens, thereby affording Dscam1 an immune receptor function. It was then discovered that upon homophilic binding, Ig5–Ig8 also form a turn in the protein but in the opposite direction to Ig1–I Ig4, which means that Ig1–Ig8 together make up a serpentine or “S” shape, binding homophilically in an antiparallel manner (25). Similar to Ig2 and Ig3, it is not known whether Ig7 is involved in heterophilic binding.

### *Dscam* Diversity in Arthropods

Taking a broader phylogenetic perspective, diversity is a common theme in the *Dscam* gene family. Although to date only pancrustaceans have been found to share *Dscam1* and its extreme somatic diversification, species from two other arthropod taxa, chelicerates (e.g., ticks), and myriapods (e.g., centipedes) have evolved diversity *via* whole gene duplication and some degree of alternative splicing (Figure 1E). For example, *Dscam* gene duplication in the centipede *Strigamia maritima* genome is estimated to have led to 60–80 *Dscam* genes and in the tick *Ixodes scapularis* to between 13 and 27 (26, 27). To date, there is no evidence of arrays of duplicated exons in chelicerate or myriapod *Dscam* Ig2 or Ig3; however, mutually exclusive alternative splicing does occur in the exons encoding for Ig7 in at least one *S. maritima Dscam* gene, and duplicated exons coding for Ig7 and Ig8 were found in four *I. scapularis* genes (26). Furthermore, two *Dscam* gene subfamilies have also recently been uncovered in the Chinese scorpion *Mesobuthus martensii*; the genes are shorter than *Dscam1* but contain Ig domains that correspond to Ig7 or Ig7 and Ig8, as well as multiple tandem exon arrays (28).

### *Dscam1* and Immune Defense in Pancrustaceans

Pancrustaceans do not have the same mechanisms for acquired (adaptive) immune defenses as vertebrates, i.e., somatic generation of receptor diversity by V(D)J joining of antibody genes followed by clonal selection of antigen-specific lymphocytes (29), which underlie immunological memory. They instead rely on the evolution of diverse innate immune defenses, which share a number of conserved features with the innate defenses of vertebrates (27, 30). Nonetheless, some pancrustaceans and other non-vertebrates show evidence of a phenomenon similar to immune memory, termed “immune priming” (31), and they can also somatically generate a limited amount of receptor diversity by alternative splicing [e.g., Ref. (32)], albeit that this diversity is many orders of magnitude lower than in vertebrates.

The link between Dscam1 and pancrustacean immunity has been extensively reviewed in the last few years and we refer readers to the following reviews for more details (6–8, 10). Here we briefly describe evidence linking Dscam1 to the immune-function hypotheses that have been proposed. Early studies on Dscam1 in *D. melanogaster* (12) hypothesized that it may function as a signaling receptor or coreceptor during phagocytosis and potentially as an opsonin [i.e., bind to the surface of a pathogen, facilitating its phagocytosis (29)]. Following this, work on *A. gambiae* also suggested the hypothesis that Dscam1 could act as a hypervariable pattern-recognition receptor for the immune system (14). Consistent with Dscam1 playing a role as an opsonin, a shorter soluble Dscam1 protein was found in S2 cell line-conditioned medium and also in haemolymph serum (12). Furthermore, Dscam1 in the shrimps *L. vannamei* and *Penaeus monodon* and the Chinese mitten crab *Eriocheir sinensis* lacks the transmembrane domain and cytoplasmic tail and has been suggested to be secreted directly into the haemolymph (33–35). It was also shown that recombinantly expressed Dscam1 protein binds to pathogens [(12), (36 this article has since been retracted), (37)].

Reducing Dscam1 *via* RNA interference (RNAi), or mutation, or antibody blocking of Dscam1 function, lead to the reduced phagocytosis of dead bacteria (12, 14), and to the hypothesis that Dscam1 acts as a phagocytosis receptor. The membrane-bound protein has been hypothesized to interact directly with the bacteria or it could interact with an opsonizing Dscam1 that has already bound to a pathogen (6, 38). Dong et al. (14) also showed that after *A. gambiae* infection with bacteria, a fungus, or protozoan parasites, *Dscam1* exon 4 produces distinct mRNA splice variants in response to each antigen (exons 6 and 10 were not tested). Dscam1 has generated intense interest in the field of pancrustacean (ecological) immunology largely because it was hypothesized that the somatic diversity generated by this gene has a function in the recognition of diverse parasite and pathogen antigens. However, we are far from understanding whether, and if so how, this might be the case.

### Our Aims

In this Hypothesis and Theory article, we bring together the ideas of six researchers who have contributed toward our current knowledge on Dscam1 in immune defense. Through our responses to seven questions, we discuss different perspectives and hypotheses on what the evidence tells us so far and our ideas for future progress on this controversial topic.

#### Question 1. Looking Back at 10 years of Research on a Potential Immune Function of Dscam1, Do You Think That Dscam1 Has a Role in Pancrustacean Immunity?

##### Daniela Brites and Louis Du Pasquier

Yes. The main reason why we think that Dscam1 has an immunological role is the fact that the diversity of its repertoires (splice variants) that are expressed in cells of the nervous system and in cells involved in immunity (fat body cells and equivalent and hemocytes) are different. Dscam1 might, therefore, fulfill functions that are specific to each of these systems, rather than have a single general purpose, which would have been suggested by identical repertoires in both tissues. Differences in exon expression patterns between the nervous and immune systems have been observed both in *Drosophila* and in *Daphnia* (12, 39). There is a lower diversity in the immune system than in the nervous system. This argues that the two repertoires are under different selection pressures and/or constraints. This restrictive evaluation may look a little provocative, but indeed the role of Dscam1 in immunity is still mysterious. As many reviews written recently point out, the situation remains unclear, with pros and cons (6, 7, 10, 40). There are too many contradictory reports concerning: (1) Dscam1 expression, whether monitored at the RNA level by PCR or at the protein level in binding assays or Western blots (up, down regulation or no change following stimulation); (2) the immunological specificity of its isoforms and the amplification of selected isoforms that has not been reproduced or convincingly demonstrated following exposure to parasites or other antigens; (3) its role as a phagocytic receptor; (4) the mode of signaling suggested by the composition of its cytoplasmic segment. The situation is complicated by the fact that *Dscam1* in pancrustaceans is not encoded in a uniform way (26). There are major differences in gene numbers and in types of alternative splicing from chelicerates to pancrustaceans (28). Even within pancrustaceans there could be room for differences in the mode of expression (e.g., importance of the soluble form) resulting in modulations of Dscam1 role in immunity.

##### Yuemei Dong

Dscam1 in insects was first characterized as a highly diverse axon guidance molecule in the neuron system of fruit flies (2, 4, 5). In the past decade, the studies of Dscam1 in mosquitoes and other pancrustaceans have established it as an essential hyper-variable pattern recognition receptor (PRR) of the innate immune system, mainly contributed by the extraordinary splice form generation at the molecular level (12, 14, 36, 37, 39).

##### Han-Ching Wang

As a crustacean immunologist, I think there is now considerable evidence to suggest that Dscam1 might be involved in immunity against non-self molecules in long-lived crustaceans such as shrimp. Dscam1 shows a typical fast (2–6 h) non-specific immune response to pathogen-associated molecular patterns (PAMPs) such as lipopolysaccharide (LPS) and beta-1,3-glucan (41, 42), but unlike most innate immune factors, Dscam1 is not always induced immediately after immune stimulation. Instead, viruses and bacteria usually take more than 24 h to induce elevated Dscam1 levels (37, 43, 44). In crayfish, this increased expression usually reaches a maximum after 5 days and then falls back to baseline levels (44). However, overall expression levels are not the only indication of Dscam1’s role in immunity, and it now appears that the correct combination of Dscam1 isoforms might be more important. For instance, we have found that some of the pathogen-induced Dscam1 isoforms induced after challenge with a particular pathogen show significantly greater binding ability to that same pathogen (37). We have also found that the haemolymph taken from “super-survivor” crayfish within 1 month of white spot syndrome virus (WSSV) challenge can provide protection to other animals against the same virus (44). Furthermore, when the Dscam1 in this haemolymph is blocked, this protection is lost (44). At the very least, it therefore seems that crayfish Dscam1 shows an ability to support an extended, specific anti-virus immune response.

##### Sophie A. O. Armitage and Joachim Kurtz

It is clear that Dscam1 is involved in immune defense in some contexts in some insects and crustaceans. However, is difficult with the current data to determine exactly what this role is, how important it is, and the generality of its importance (7, 10). For example, immune gene expression (total or alternatively spliced variants) after exposure to a pathogen or parasite shows varied results across studies [reviewed in Ref. (7, 10)], and gene knockdown can reduce survival after infection (14), but it can also have no effect on survival (40). Furthermore, some host–pathogen interactions seem to provide more convincing evidence [e.g., *A. gambiae* and *Plasmodium* spp. (14, 45)] than others (40) for a role of Dscam1 in immunity. *Dscam1* might not necessarily play an important role in all taxa, but instead be an “add-on” to immunity, where described phenomena are the side effects of e.g., altered hemocyte behavior. It is also worth bearing in mind that only a tiny fraction of the extremely speciose Pancrustacea have been examined to date. We do not know whether *Dscam1* publishing bias exists, in terms of an under-representation of “negative results,” but should unpublished data be sitting on someone’s hard drive it could be helpful to share this information to unravel the conditions under which *Dscam1* does or does not respond in an immunological context.

#### Question 2. Dscam1 Was Hypothesized to Produce the Large Number of Variable Receptors Needed for Specificity in the Immune Response, in Some Ways Analogous to Antibodies in Vertebrates. Do You Think That Dscam1 Is Indeed the Equivalent of Antibodies As Specific Immune Receptors?

##### Daniela Brites and Louis Du Pasquier

No. We think that there is a lot of confusion around the Dscam1 analogy with antibodies. With respect to Dscam1’s somatically acquired diversity we think that the analogy with antibody diversity has been over-emphasized even though a warning had been formulated at the very beginning (46). One has had the tendency to compare apple and oranges. To be analogous to antibodies, Dscam1 isoforms, specific to a pathogen epitope, should be secreted by some clones of uncommitted hemocytes that resulted from the stimulation of a precursor cell expressing the relevant Dscam1 specificity. This is what happens in the adaptive immune system of vertebrates where specifically stimulated uncommitted B lymphocytes, the DNA of which has been somatically modified to encode a single receptor specificity per cell, proliferate (i.e., generating a clone) and differentiate into secreting plasma-cells that release large amounts of one antibody. Today, so far, nothing of the above applies to Dscam1. Since *Dscam1* variability is produced at the RNA level, it is not inheritable in the progenies of cells, would those cells where splicing occurred divide. But anyway there are so far no reported specifically induced proliferative responses of Dscam1 producing cells. Unlike what has been proposed (47) there is no clonal amplification of the cells producing Dscam1. In addition adult flies do not produce new cells from the hematopoietic organs. However, one should be careful not to generalize from a single species or stage. In fact in the light of the interesting recent observations of transdetermination and proliferation of hemocyte lineages reported in *D. melanogaster* larvae, it might become interesting to follow Dscam1 expression on those cells even though no information on their clonality is available (48). One speaks carefully of “demand adapted increase in hemocyte proliferation.” Increases in cell numbers in parasitized flies have been reported as being due to proliferative response but an increase from 0 to 1,000 cells in 6 h cannot be due to a simple proliferation. There is here something new to investigate. There have been many examples of induction of *Dscam1* gene expression after some “antigenic” exposure (Membrane form? Soluble form? This is not always specified). In addition, significant increases in Dscam1 diversity were observed in parasite-exposed mosquitoes (49). Increasing diversity after immunization does not make Dscam1 a likely analog of antibodies. Indeed, following immunization one would rather see the amplification of one or two useful variants with some specificity like in antibody responses. Increasing diversity means lowering the concentration of single isoforms and therefore offering minimal chances for profiting from a special binding property. But we may simply not understand the mode of action of Dscam1. Since apparently a single cell expresses more than one Dscam1 isotype (a profound difference compared to uncommitted lymphocytes) the best that can be produced is a “shot gun” of unrelated Dscam1 molecules (8). This leads us to an issue that has been often neglected: the concentration of each isoform either on the cell surface in the hemocyte population, or in the biological fluids. If one assumes that one variant of Dscam1 has a better avidity for its ligand, how can this advantage be exploited? It is difficult to imagine the utility of a single variant diluted in the middle of thousands of other forms, so there is a need for selection and amplification steps. Those are difficult to conceive in a system where diversification is due to mutually exclusive alternative splicing and without specific cell proliferation. Therefore, Dscam1 diversity might have a function other than being a repertoire of antigen reactive molecules.

##### Yuemei Dong

Lacking in vertebrate antibodies, insects rely on relatively small numbers of PRRs to combat various pathogens during their complex life cycles, which for a long time lead researchers to believe that the immune system in the invertebrates is not as sophisticated as its counterpart in the vertebrates (50–53). The genetic expansion of *Dscam1* and its ability to generate enormous pathogen specific receptors through immune responsive alternative splicing have equipped insects with a similar level of complexity at the molecular level, and thereby generate astounding analogs to antibodies. The rapid progress of *Dscam1* research in immunity has marked its role and importance in insect immunity, a ground-breaking contribution that blurs the classical strict clarification between innate and adaptive immunity (12, 14, 39, 53). Innate immunity used to be defined as being dependent on germ line encoded receptors, rather than recombination of somatically expressed antibodies, therefore *Dscam1*’s role in immunity fits the strict definition of innate immunity as it is germ line encoded, but not in the sense of the definition as *Dscam1* produces immune responsive splice forms. The vast diversity of the antibody system is clearly adaptive, hypothetically *Dscam1* also seems adaptive when considering it can produce tens of thousands potential splice variants.

##### Han-Ching Wang

The three hallmarks of acquired immunity are immune diversity, immune specificity, and immune memory (51). Mammalian antibody-based immune systems have all of these abilities; however, although there is evidence to suggest that Dscam1 is also able to support all of these functions, Dscam1 would have to provide these functionalities *via* different mechanisms than those used by antibodies. *Dscam1* is capable of immune diversity and immune specificity through alternative RNA splicing (2, 14, 33, 35, 39, 44, 45). However, after pathogen challenge, we still do not know whether immune cells in pancrustaceans are somehow able to actively design the particular alternative exons that show the ability to bind to the pathogen, or alternatively, whether populations of pathogen-induced specific Dscam1 isoforms are created either through positive selection or by the same kind of negative selection mechanism that is used in vertebrate adaptive immune systems. Another curious similarity between Dscam1 and antibodies is that whereas antibody diversity/specificity is achieved by combinations of three gene segments, V(D)J, Dscam1 hypervariability is achieved *via* three variable exon regions, Ig2/Ig3/Ig7. Furthermore, since most Dscam1 studies have so far focused primarily on particular variants rather than the whole Ig2/Ig3/Ig7 Dscam1 combination, we might therefore be underestimating the potential immune specificity of Dscam1. Maintenance of the appropriate Dscam1 populations is another problem, and immune memory in pancrustaceans is still an open question. In antibody-based mammalian immune systems, memory is achieved by somatic changes, but in pancrustaceans, to date, no convincing model of immune memory has yet been established.

##### Joachim Kurtz

My answer depends on what we mean when we say “equivalent.” The original view that Dscam1 might function like antibodies [e.g., Ref. (38, 47, 50)] was probably a bit too optimistic, since several crucial elements could as of yet not be demonstrated and are maybe unlikely to exist: there seems to be no clonal amplification of the cells that produce the “right” isoforms, and maybe no receptor for Dscam1 that could serve a similar role as the Fc receptor for a hypothetical opsonin-like function of Dscam1. Having said this, we should still be aware that being “equivalent” does not mean that everything has to be similar, and if we search for an equivalent system to produce somatically diversified receptors, then *Dscam1* is still “alive and kicking.” It is reasonable to assume that some form of somatic diversification is needed to produce a sufficiently large pathogen receptor repertoire that would be needed for specificity in discrimination among a large number of potential antigens. As of yet, we have only very limited evidence that pancrustaceans are actually able to achieve such a very high level of specificity in their pathogen and parasite defenses [e.g., Ref. (54)]. But if they are able, *Dscam1* is currently the only system known for pancrustaceans that could at least theoretically provide the needed receptor diversity. However, critical tests of the involvement of Dscam1 in the specificity of immune reactions are still lacking.

##### Sophie A. O. Armitage

Through combinatorial diversification of vertebrate variable, diversity and joining gene segments (V(D)J) millions of combinations can be produced, and this number is in the order of billions of antibody molecules as a result of junctional diversification and somatic hypermutation (55). Dscam1, on the other hand, shows many orders of magnitude less diversity than vertebrate antibodies. Through ultra-deep sequencing of *D. melanogaster Dscam1* mRNA using next generation sequencing, Sun et al. (13) detected 18,496 of the possible 19,008 isoform combinations for exons 4, 6, and 9. However, *D. melanogaster* fat body and hemocytes do not express the full range of particularly the exon 9 cluster (11, 12), which could considerably reduce the total isoform estimation. Therefore, in addition to the above responses to this question, in terms of variation, *Dscam1* does not produce diversity that is equivalent to that produced by antibodies as specific immune receptors.

#### Question 3. Next to Specificity, Remembering Is a Key Aspect of Immune Memory. Could This Be Achieved with Dscam1?

##### Daniela Brites and Louis Du Pasquier

No, according to our conservative concept of memory! Memory, in an immunological sense, demands clonal amplification and storage of specialized cells. It implies a reactivation of those cells after the initial antibody response has been down regulated (anamnestic response). This does not happen in any invertebrate and more specifically it does not happen in the Dscam1 case. However, if some soluble form with specificity persists, the protection that it may confer can persist: it will be called memory by some but not by classical immunologists who see then a persisting on-going response or the long survival of a protecting agent and not the proper “recall” that characterizes memory responses.

##### Yuemei Dong

Evolution might have taken different routes to achieve functional similarities with Dscam1 splice clouds in the invertebrates’ and antibodies in the vertebrates’ immunity. Given the two major features of adaptive immunity, immune specificity and memory, much of the work about Dscam1’s role in adaptive-resembling immunity was focused on addressing the pathogen recognition diversities and specificities. Quite some studies have shown that past infections influence insects’ humoral and cellular immune system thereby protecting the host from the second and the following pathogenic infections (53, 56–62). So-called immune priming or trained immunity has now been demonstrated in a wide range of pancrustacean species. However, with the currently available data, it still remains to be demonstrated that recognition specificities mediated by Dscam1 splice variant repertoires have memory.

##### Han-Ching Wang

Although there is increasing evidence to suggest that immune memory occurs in pancrustaceans, the underlying molecular mechanism is still an open question. In pancrustaceans, it has long been clear that somatically generated immune factors, such as lectins and proPO-related proteins, could not account for immune specificity or immune memory. When *Dscam1* was discovered, it seemed to have great potential to support these special immune responses. Initially, however, most *Dscam1* studies were performed in short-lived pancrustaceans, which made it difficult to investigate its role in immune memory. In a recent, as of yet unpublished study, we challenged 200–300 long-lived crayfish twice with WSSV, with the second challenge made 14 days after the first. We then used gene cloning to determine the expressed combinations of Ig2–Ig3 in the Dscam1 populations in collected hemocyte samples. In the crayfish that survived both challenges, some Dscam1 isoforms with particular Ig2–Ig3 combinations showed a good binding affinity with WSSV. Furthermore, these isoforms appeared after the first challenge and they increased in quantity after the second challenge. This result is consistent with the idea that there might be meaningful selection and maintained expression of particular *Dscam1* exons. Unfortunately, there were also complications: first, the expression pattern was not seen in every surviving crayfish, and second, each surviving crayfish produced different Dscam1 isoforms.

##### Sophie A. O. Armitage and Joachim Kurtz

We here consider a phenomenological definition of immune memory, which has been called “immune priming” in invertebrates and can be described as “the ability of an immune system to store or simply use the information on a previously encountered antigen or parasite, upon secondary exposure” (31), rather than considering a mechanistic definition invoking the acquired immune system. Since a review (7), where we discussed the absence of empirical data on the hypothesis that Dscam1 is involved in immune priming, there are at least two published studies on this topic (63, 64). The latter study found no change in *Dscam1* gene expression in a transgenerational immune priming study. However, Fu et al. (63) found that shrimp that had fed on bacterial spores harboring a WSSV protein, and then received siRNA to knockdown *Dscam1*, were less phagocytically active and had lower survival after a subsequent exposure to WSSV compared to shrimp that had also been primed with WSSV protein but did not receive *Dscam1* siRNA. This study would support the hypothesis that *Dscam1* has some involvement in immune priming, but we note that the expression of individual splice variants was not tested. If we imagine the hypothesis that *Dscam1* splice-variants are specific for a particular pathogen [this was not tested by Fu et al. (63)] it is difficult to conceive the mechanism by which *Dscam1* could “remember” aspects of previously encountered antigens. Variability in the *Dscam1* isoforms comes from somatically generated mRNA *via* mutually exclusive alternative splicing, therefore there would need to be some mechanism by which splicing patterns can be reproduced. Alternatively the variation in *Dscam1* may not be important for immune priming, it is just the presence or absence (reduction) of the protein that affects the phenomenon.

#### Question 4. What Alternatives Are There to an Antibody-Like Function of Dscam1 in Pancrustacean Immune Systems?

##### Daniela Brites and Louis Du Pasquier

To sum up, *Dscam1* diversity as a whole seems to be the selected feature (diversity for diversity’s sake) and we see it best exploited in the nervous system i.e., to specify cell identity. “Thus, the Dscam1 repertoire of each cell is different from those of its neighbors, providing a potential mechanism for generating unique cell identity in the nervous system and elsewhere” (11). We therefore suggest that in a manner analogous to what it does in the nervous system, Dscam1 on hemocytes might specify hemocyte identity, using homologous interactions in the way proposed for neurons (20) (see below paragraph 7 a suggestion for a method). Other functions could be inferred from understanding better the signaling capacities of the molecule.

##### Han-Ching Wang

In crustaceans, a pathogen can induce “antibody-like” Dscam1 isoforms that show specific binding ability to the invading pathogen. In shrimp, but not yet in crayfish, we have also observed “super Dscam1 isoforms” that have a wider binding ability to a range of bacteria and viruses (37). We have also seen that while a whole intact pathogen takes ~24 h to induce *Dscam1* expression (37, 43, 44), challenge with pathogen-associated molecular patterns (PAMPs), such as lipopolysaccharides (LPS) and beta-1-3-glucan and peptidoglycan (PG), induce *Dscam1* expression within just a few hours, after which *Dscam1* expression levels then decline (41, 42). These findings suggest that, as with other innate, non-hypervariable, crustacean immune factors, *Dscam1* can also be triggered even without any antigen-specific recognition. Taking all of these results together, it is tempting to propose that it might be the “super Dscam1 isoforms” that are responsible for this rapid, non-specific immune response (37). A corollary of this proposal is that *Dscam1* might therefore be regulated by at least two molecular mechanisms: one involved in the regulation of *Dscam1* expression; the other involved in the regulation of alternative splicing to generate specific Dscam1 isoforms.

##### Joachim Kurtz

It is important to note that one of the “beauties” of the antibody system lies within the fact that antibodies are at the same time specific receptors and powerful effectors, such that the specificity of recognition is directly linked to the defensive function. However, this does not need to be the case for other immune molecules and provides the alternative that in the case of *Dscam1*, there might well be a function as a pathogen receptor (the studies demonstrating binding to pathogens support this view), but not as an effector (the mixed results regarding expression changes upon infection and the relatively low expression level of *Dscam1* in the immune system suggest this). The receptor function could be somewhat similar to the role of *Dscam1* in the nervous system. Hemocytes interact with one another when they encapsulate a pathogen or close a wound, while such interactions could in the absence of an insult be blocked by Dscam1, just as Dscam1 homophilic binding blocks neuronal self-interactions. In this context it is intriguing that the parts of Dscam1 that are responsible for homophilic interactions differ from the potentially pathogen-binding parts (24), so that both functions could co-occur. More generally, neuro-immunological feedbacks could be involved and link the neuronal function of *Dscam1* to its immune function. Such feedbacks are for example known for the regulation of immune genes by the internal clock (65) and it would be worth exploring an immune regulation role for *Dscam1*.

##### Sophie A. O. Armitage

Dscam1 has been proposed to act as a hypervariable PRR, a co-receptor during phagocytosis and an opsonin. As mentioned above, in addition to, or instead of, directly interacting with antigens, cell surface expressed Dscam1 might be important for host cell–cell interactions, be these from hemocyte to hemocyte, or hemocyte to fat body/nervous system/other cell. If these interactions were in the form of homophilic binding, and if each cell has a restricted repertoire of isoforms, then the frequency of Dscam1 homophilic binding between different cells would likely be low. Furthermore, if Dscam1 interacts with pathogens, one could hypothesize that it also interacts with non-pathogenic microbiota found within the host. There are indications that Dscam1 influences microbiota, more specifically bacteria, in *A. gambiae*: knockdown of *Dscam1* increased bacteria in the haemolymph (14) and overexpression of a particular *Dscam1* variant reduced bacteria in the gut (45). In contrast, in the small brown planthopper, *Laodelphas striatellus*, the titer of an extracellular symbiotic bacterium was unaffected by *Dscam1* knockdown, and the titers of an endosymbiotic bacterium, *Wolbachia*, and the rice stripe virus were even decreased after knockdown (66). It is not clear why intracellular passengers would be affected by the knockdown of a cell adhesion molecule on the surface of the cell, is it a direct effect of the reduction in Dscam1 or does knockdown negatively affect the host cells or their behavior in some way, so reducing survival for intracellular passengers? These are speculations, but it will be interesting to see whether other host–microbe interactions are influenced by *Dscam1*.

#### Question 5. What Is the Meaning of Dscam1 Genetic Diversity?

##### Daniela Brites and Louis Du Pasquier

Comparative analysis of *Dscam1* in different arthropod groups has shown that two mechanisms of generating *Dscam1* diversity have evolved independently; massive whole-gene duplications in basal arthropods and the refined mutually exclusive alternative splicing of duplicated exons in the *Dscam1* of pancrustaceans. The ability to generate *Dscam1* diversity seems thus to have been positively selected in the evolutionary history of arthropods. Perhaps because diversity provided means of specifying cell identity (e.g., in hemocytes which are important mediators of embryonic development). We still know very little about *Dscam1* in basal arthropods, however, the evolution of pancrustacean *Dscam1* is well studied. We can conclude that in contrast to the constitutively expressed domains of *Dscam1* which are highly conserved, the alternative domains encoded by the alternative exons are highly diverse across pancrustaceans. If providing cell identity has been the most important driver of *Dscam1* diversity and that already happened in the most recent common ancestor of pancrustaceans, why would each group of pancrustaceans have evolved its own set of alternative exons? Could that be driven by an additional role in immunity? Then there is the question of *Dscam1*’s polymorphism within species, and what we can learn from it. In *Dscam1* polymorphism can be understood *sensu lato* both as the variants generated within an individual *via* alternative splicing of duplicated exons of *Dscam1*, and as polymorphism at the population level caused by mutations accumulated in orthologous exons in different individuals. The first source of polymorphism we have touched upon already and we would briefly like to mention what we have learnt from studying *Dscam1*’s allelic polymorphism. The regions of the variable domains that are not involved in the homophilic binding of the molecule (so-called epitope II) are more diverse (at the population level) than the regions involved in homophilic binding. Why are they more diverse? Could these variants be important for antigen recognition? Population genetic tests did not provide solid evidence supporting that these variants are maintained in the population because of antigen recognition, however the power of these analyses was low (67). The question of whether epitope II could be involved in binding to antigens therefore still remains open and should be tested experimentally.

##### Han-Ching Wang

Although Dscam is a ubiquitous protein that can be found in various animal species, such as mammals, fish, mollusks and arthropods, I would like to discuss its genetic diversity solely in terms of arthropod Dscams. Curiously the ancestral hypervariable *Dscam1* gene is only found in the pancrustaceans, while other arthropods have non-hypervariable Dscam-like genes (68). This situation presumably arose due to independent gene duplication and diversification events that in turn would be driven by their adaptive value in the evolution of the *Dscam1* gene family during Arthropoda evolution (68). It is very likely that this evolutionary pressure depended on the functional requirements of the arthropod’s nervous system and/or its putative immune system, and in this content, it is important to note that the genetic diversity of *Dscam1* depends on both its extracellular region and its intracellular region. The hypervariable Dscam1 extracellular region is used for axonal guidance during neuronal development and also provides a mechanism that might, at least potentially, be used for pathogen recognition (4). But the intracellular Dscam1 cytoplasmic tails also show an interesting divergence: for instance, although there is a high homology between insect *Dscam1*s, the crustaceans have evolved quite differently, with variable cytoplasmic tails in shrimp (34), and a unique tail-less form of Dscam1 in shrimp and crab (33–35). Furthermore, the secreted insect Dscam1 is generated from membrane-bound Dscam1 by a shedding process (47), whereas the more long-lived crustaceans express the tail-less Dscam1 directly through alternative splicing (33–35). The way that this tail-less Dscam1 is directly expressed bears a thought-provoking resemblance to the way that secreted IgM antibodies are expressed in mammals. While this might simply be a coincidence, it might also be a form of convergent evolution that reflects the importance that immune memory should have to a long-lived arthropod (i.e., a crustacean) as opposed to arthropods with shorter lifespans (e.g., most insects).

##### Joachim Kurtz

Generally, genetic diversity can come in different flavors: as diversity in the population (i.e., polymorphism) and as diversity within each individual. Accordingly, these different types of diversity could have different meanings: diversity in the population could have arisen from the processes of gene duplication and mutation and could be maintained by negative frequency-dependent selection, for example by parasites. Diversity in the individual might further be increased by somatic diversification processes, such as alternative splicing in the case of *Dscam1*. Its meaning could be diversity just for its own sake, which seems to be what is going on for *Dscam1* in the nervous system, so as to enable neuron self/non-self discrimination. Alternatively, its meaning could be to produce immune repertoire diversity so as to recognize diverse parasitic antigens. It is interesting to compare with other systems [for review see Ref. (31)], where immune diversity sometimes stems from massive diversification in the germ-line, such as in the case of V region-containing chitin binding proteins (VCBPs) in amphioxus, while it mainly comes from somatic diversification processes in other systems, such as the vertebrate antibodies and maybe the mollusks’ fibrinogen-related proteins (FREPs) and the Sp185/333 proteins of sea urchins. For *Dscam1*, it is still difficult to say which of these potential “meanings” of genetic diversity is most relevant.

##### Sophie A. O. Armitage

Diversity in *Dscam1*, and more generally in the *Dscam* gene family, operates at different levels. Starting with a broader perspective, *Dscam1* paralogs have been described for insects (39, 68, 69) and at least one crustacean (27), showing that diversity exists at the level of whole gene duplications. For example in addition to *Dscam1*, the *D. melanogaster* genome contains *Dscam2, Dscam3*, and *Dscam4*, of which only *Dscam2* has (two) alternatively spliced exons in Ig7 (70). Narrowing our perspective to just the *Dscam1* gene, diversity is found across orthologs in terms the number of alternatively spliced exons that have evolved within each of the alternatively spliced exon cassettes found in a species. For example, from the lower diversity *Dscam1* in *Daphnia magna* [8, 24, and 17 alternatively spliced exons in Ig2, Ig3, and Ig7, respectively (39)] to higher diversity in *Anopheles gambiae* [14, 30, and 38, respectively (71)]. Reconstructing the evolutionary history of alternatively spliced exons across pancrustacean species with confidence proved difficult, probably because of the relatively short exons and long evolutionary timescale studies (68). It was possible to infer orthologs of most of the Ig2 and Ig7 variants between comparatively closely related species, i.e., *D. melanogaster* and *D. mojavensis*; but this was more difficult for Ig3, indicating more duplication or deletion events and resulting in a faster accumulation of diversity in this cluster of exons compared to Ig2 and Ig7 (68). In contrast, the amino acid sequences of the non-alternatively spliced regions in *Dscam1* orthologs show greater conservation (12). To zoom into the last level, we know relatively little about within-species diversity in terms of polymorphisms in the conserved or alternatively spliced regions of *Dscam1* [but see Ref. (67, 72)]. It has been hypothesized that because diversity within individuals is generated somatically, that one might not expect to find strong signatures of selection in the alternatively spliced exons (7, 10).

#### Question 6. What Was the Main Factor Driving the Evolution of Diversity in Dscam1—The Nervous or Immune System or Even Something Else?

##### Han-Ching Wang

It is interesting to note that, just like Dscam, a number of immune factors/receptors also play an important role in the neuronal system, and in fact there is increasing evidence that both systems share several mechanisms and have similar physical properties. Currently, however, it is still too early to say whether *Dscam1* diversity evolved dependently or independently of the nervous system because work in *Dscam1* neuroimmunology is still in its infancy in insects and has not even begun in long-lived crustaceans. Even so, based on current knowledge, I tend to believe that the diversity in *Dscam1* must on some level be driven by immune-related evolutionary pressure. First, at least in *D. melanogaster*, the ways that *Dscam1* alternative exons are used in neural cells and immune cells are different (12), suggesting that the regulation and exon selection of *Dscam1* alternative splicing may be mediated by different mechanisms. Second, in shrimp, our experimental data showed that recombinant Dscam1 proteins containing various Ig2/Ig3 combinations bound more strongly to natural shrimp pathogens (such as *Vibrio harveyi* and WSSV) than to other bacteria (*Escherichia coli* and *Staphylococcus aureus*) (37). This suggested that host–parasite coevolution may have occurred in a way that contributed to *Dscam1*’s hypervariability in immunity.

##### Joachim Kurtz

We can only speculate here, but when we consider that outside of the Pancrustacea, Dscam’s function seems to be only in the brain, then it is more likely that Dscam’s role in the nervous system predates its function in the immune system. So let us assume there was an ancient function of Dscam in the brain, what could have driven the evolution of diversity? It is not unlikely that there was negative frequency-dependent selection, because a rare isoform has the advantage that it offers a higher value for the function to discriminate neurons. For a rare isoform, few other neurons will express the same isoform. However, a novel isoform also bears the risk of potentially harmful self-reactivity with any other pattern in the organism, leading to selection against self-reactive isoforms, i.e., self-reacting Dscam isoforms would be purged from the isoform “pool.” As a result, but still predating any immune function, we could imagine that with Dscam a molecular system has evolved that represents “non-self.” This could then have been a preadaptation (i.e., an evolutionary “exaptation”) for a system that would allow for non-self recognition also outside of the nervous system, i.e., a potential pathogen recognition system could have emerged. This way, an immune function might have followed from a more ancient nervous system function. Once there, selection pressures from the immune system would kick in and lead to further diversification. And finally, there is yet another possible initial driving factor for the evolution of diversity: to enable histocompatibility reactions within the species, i.e., allorecognition [see, e.g., Ref. (73, 74)], which for example explains the diversity at the *fuhc* locus in the ascidian *Botryllus*, where the *fester* gene also shows quite extensive alternative splicing [(75); for review see Ref. (76)]. Allorecognition systems have likely evolved in taxa where chimerism is a relevant problem, such as colonial invertebrates, where there is in particular the risk of germ-line parasitism. It would thus be interesting to find out whether or not chimerism might have played a role in those arthropods that initially diversified *Dscam1*.

##### Sophie A. O. Armitage

This question is difficult to answer with our current knowledge. Considerable data exists describing the function of *Dscam1* in the nervous system of *D. melanogaster* [reviewed in Ref. (5, 9)]. *Dscam1* mRNA is expressed in the brain of other species of Pancrustacea [e.g., *Daphnia* (39)], but our knowledge of how Dscam1 functions in the nervous system of these species is less well understood. Studies focusing on the function of *Dscam1* in basal pancrustacean species might help to elucidate the selection pressure that maintains current diversity. Perhaps diversity in Ig7, which can also be found in non-*Dscam1* genes, initially evolved in response to different cues to those that resulted in diversity in Ig2 and Ig3? As detailed above, the Dscam gene family in arthropods is highly diverse; what were the selection pressures that lead to diversification not only of *Dscam1*, but also of the *Dscam* gene family in general? Was this the same selection pressure? We know that some of the non-highly diversified *Dscam* genes function in the nervous system [e.g., Ref. (69, 70, 77)], do these genes also play immune roles? Do taxa that are evolutionarily basal to arthropods, e.g., Onychophora and Tardigrada, have *Dscam* homologs, if so are they diversified and what are the functions of these genes?

#### Question 7. What Are the Future Perspectives for Studies on Dscam1 in Immunity? (Including What Essential Experiments or Approaches Are Missing That Would Help Our Understanding of Dscam1 in Immunity?)

##### Daniela Brites and Louis Du Pasquier

(1) Make more reagents. Raise more monoclonal antibodies to follow and play with expression in different species. (2) Repertoire analysis. How does restriction of Dscam1 isoform per single cell work? How stable is it? Similarly to what happens in neurons, does Dscam1 splicing vary overtime in one immune cell [e.g., *Daphnia* (39)]? Use NGS for complete repertoire analysis over time after antigenic stimulation of all hemocytes including the especially interesting subsets recently discovered (78). (3) Cellular assays. Try plaque forming cell assays or ELISPOT assays to see whether there is real secretion vs. shedding by hemocytes or other cells. The *D. melanogaster* S2 cells that have been studied (48) produce perhaps a reduced repertoire of 15–50 different Dscam1 molecular categories/cells but are far from being uncommitted. Proliferating in artificial conditions *in vitro*, S2 cells are not the equivalent of *in vivo* lymphocytes, but at least they are derived from the macrophage-like cell type of *D. melanogaster* and divide every 24 h at 26–28°C. This might still provide an *in vitro* model for understanding Dscam1 signaling, stability of expression, and properties of progeny cells within the hematopoietic tissues. (4) Signaling. What are the consequences of ligand/receptor interactions? The signaling pathways that are known for Dscam1 are still controversial (11). A possible relationship with the cytoskeleton has been suggested, which could be compatible with a role in phagocytosis and/or in cell movement. *Dscam1* mutants should help elucidating this aspect. How does *Dscam1* induction (upregulation) work in the fat body and hemocytes? Is it *via* direct stimulation by Dscam1 receptors themselves, or *via* a cytokine? Or is it *via* Toll, JAK, or Imd pathways? Are coreceptors involved? (5) Exploit more *Dscam1* mutants in immunological experiments. Test the alternative hypothesis mentioned above (i.e., see 4) in *Dscam1* mutants. Migration of hemocytes can be monitored beautifully in *D. melanogaster* [(79), this article has since been withdrawn]. Following mechanical disturbance hemocytes change location and return to their original position within 45 min. If Dscam1 plays a role in controlling migration of hemocytes, *Dscam1* mutants should show differences in relocation after disturbance, the prediction being that the cells would not return properly to their location. But perhaps the pattern of hemocytes distribution in mutants would be abnormal even without disturbance! (6) Specificity of binding. One should explore the binding properties of Dscam1 proteins to heterologous ligands to confirm its potential as a receptor or an effector. In addition, study the precise binding properties of Dscam1, with proteins encoded for by different exon combinations, to determine the specificity of binding heterologous ligands (if any). Go back to testing further the epitope I–epitope II hypothesis, with the *in vitro* production of Dscam1 molecules, similarly to what was done by Watson et al. (80). (7) Comparative functional approaches. Study the role of Dscam in basal arthropods. Are the functions of the Dscam1 molecules all analogous to each other? Are they redundant? Compare again hemocytes versus other cells and investigate the presence of soluble forms.

##### Yuemei Dong

Many questions remain to be answered, such as how many splice variants (or groups of variants, so called “Dscam1 clouds”) are produced uniquely or whether there is a continuous range. Moreover, the essential questions regarding the stability of the pathogen specific Dscam1 isoform repertoires after selection, and whether the expression of Dscam1 clouds in the renewing population are regulated, remain to be addressed.

##### Han-Ching Wang

There are still many missing pieces in the puzzle of *Dscam1*-mediated immunity, even in terms of Dscam1’s general properties. For instance, we still do not have a complete picture of *Dscam1*’s response at the mRNA level and protein level after one or multiple stimulations with various immune stimulators. Part of the difficulty in *Dscam1* research is due to the fact that it cannot easily be silenced *in vivo* in long-lived crustaceans, such as shrimp and crayfish (unpublished data). Clearly, there is a need to develop an alternative *in vivo* system to test *Dscam1* function, especially for long-term observations. As for *Dscam1*’s immune diversity, the main questions to be addressed are which factors are involved in *Dscam1* alternative splicing and which mechanisms support the generation and maintenance of the specific Dscam1 isoforms after pathogen challenge. Meanwhile, regarding Dscam1’s immune specificity, instead of just focusing on one particular highly expressed exon variant, we should investigate how the entire Ig2/Ig3/Ig7 combination is involved in specific binding with the corresponding pathogen. We would also like to know which kinds of epitopes on a particular pathogen can be recognized by the corresponding pathogen-induced Dscam1 isoforms: does Dscam1 bind with these pathogens through the recognition of general PAMPs or by recognizing particular antigens as pathogen surface proteins? Finally, the question of immune memory is perhaps the most difficult of all. Our current approach is to document the dynamics of the Dscam1 isoform population in long-lived pancrustaceans after multiple stimulations. From this, we hope to establish whether or not some specific Dscam1 isoforms are consistently present after specific pathogen stimulation. Other open questions include: which cell types might act as immune memory cells? Are the kinds of pathogen-specific Dscam1 isoforms expressed after pathogen stimulation only produced by particular cells (or cell types)? At present, we are still a long way from answering these questions. Given that penaeid shrimp culture is a global economic activity that is vulnerable to economic losses from outbreaks of viral and bacterial diseases, the study of Dscam1-mediated immunity is also of practical importance. For example, a clear understanding of the mechanism of Dscam1-mediated immunity should provide a scientific basis for optimizing shrimp vaccination strategies. We therefore believe that further research into *Dscam1* has great potential and should very much be encouraged.

##### Sophie A. O. Armitage and Joachim Kurtz

In addition to the abovementioned ideas we would add: (1) test whether epitope II indeed binds to pathogen/parasites, and if so, uncover what the specific binding partner is; are there conserved aspects of e.g., viruses, bacteria, fungi, or other parasites that are involved? (2) next, generation sequencing of mRNA alternative splicing patterns of exons 4, 6, and 9 [e.g., Ref. (81)], for example, using the *A. gambiae*—*Plasmodium* interaction or crustacean—WSSV interactions, which seem to be particularly promising to understand *Dscam1* in immunity; (3) as an extension to the previous point, applying peptide sequencing to Dscam1 after infection with a pathogen or parasite to test the variability in alternatively spliced sequences at the protein level; also determine for how long the protein persists in the haemolymph (particularly in relation to knock-down studies); (4) test whether Dscam1 is involved in specific immune memory by varying the identity of the primary and secondary pathogen/parasite in conjunction with Dscam knockdown before the primary and/or before the second pathogen/parasite exposure; and (5) it could be interesting to further characterize the influence of Dscam1 on microbiota.

## Author Contributions

JK and SA conceived the questions. SA collated the answers, wrote the introduction, and produced the figure. All authors responded to the questions and revised the manuscript.

## Conflict of Interest Statement

The authors declare that the research was conducted in the absence of any commercial or financial relationships that could be construed as a potential conflict of interest.
